# An Atypical Presentation of Posterior Reversible Encephalopathy Syndrome (PRES): Sticking to the Basics

**DOI:** 10.7759/cureus.71836

**Published:** 2024-10-19

**Authors:** Naweed Essa Ebrahim Essa, Muhammed Ameen Noushad, Prutha Chawda

**Affiliations:** 1 General Internal Medicine, University Hospitals Plymouth NHS Trust, Plymouth, GBR; 2 Neurology, University Hospitals Plymouth NHS Trust, Plymouth, GBR; 3 Radiology, University Hospitals Plymouth NHS Trust, Plymouth, GBR

**Keywords:** atypical pres, posterior fossa, posterior reversible encephalopathy syndrome (pres), posterior reversible leukoencephalopathy syndrome, reversible posterior leukoencephalopathy syndrome (rpls), severe hypertension, travel history

## Abstract

Posterior reversible encephalopathy syndrome (PRES) is a reversible clinico-radiological condition primarily affecting the occipito-parietal regions. Thalami, brainstem, and cerebellar involvement with posterior fossa oedema are rare manifestations of this condition. We present the case of a 66-year-old male with a travel history to Thailand who was found collapsed on the floor two weeks after his return. He did not have any history of neurological or systemic symptoms. A head computed tomography (CT) showed extensive posterior fossa and brainstem oedema resulting in tonsillar herniation and mild hydrocephalus. Magnetic resonance imaging (MRI) study revealed widespread symmetric T2/FLAIR changes in the supratentorial and infratentorial brain parenchyma, microhemorrhages, and florid punctate enhancement in the affected regions. After his initial investigations, the differential diagnosis included acute demyelinating encephalomyelitis (ADEM) and viral rhomboencephalitis. Blood pressure was elevated on admission and intensive care unit stay. Upon achieving blood pressure control, the patient’s clinical picture improved and a diagnosis of PRES was made.

Our case highlights how confounding factors make the diagnostic process challenging. Atypical presentations of PRES are rare but should be considered in patients with risk factors such as uncontrolled hypertension and acute neurological symptoms in the context of MRI findings of vasogenic oedema.

## Introduction

Posterior reversible encephalopathy syndrome (PRES) is a condition characterised by reversible radio-clinical features. It can have acute or subacute presentations, with symptoms such as altered mental status, visual disturbances (e.g., visual hallucinations, visual field defects, and cortical blindness), headaches, and seizures [1-2].

PRES is commonly associated with conditions such as acute renal failure, severe uncontrolled hypertension, septic shock, autoimmune disorders, and cytotoxic medications [3]. The pathophysiology of PRES is still unclear, and different theories have been postulated. There have been two primary mechanisms described in the literature. The first one states that a hyper-perfusion state, such as acute hypertension, leads to impaired cerebral autoregulation, which causes a breakdown of the blood-brain barrier and subsequent oedema. The other mechanism suggests that some predisposing conditions release Endothelin-1, Prostacyclin, and Thromboxane A2, which lead to vasospasm, ischaemia, and cerebral oedema [1,3-5]. In this case, we present a patient with an atypical presentation of PRES with brainstem involvement and significant posterior fossa oedema, who has a travel history to Thailand.

## Case presentation

A 66-year-old male who had been living in Thailand for the past three months presented with a history of feeling generally unwell for two weeks, along with nausea and vomiting. He had no history of fever, seizures, or systemic symptoms. He did not attend any hospital in Thailand and returned to the UK. He was found collapsed on the floor two weeks later. His medical history included prostate cancer, for which he had undergone radical prostatectomy and external beam radiation. He was not on any regular medication and there was no history of recreational drug use. While in Thailand, a routine check-up indicated that the patient had hypertension. However, he chose to decline treatment.

Upon examination in the emergency department, he was found to have a reduced level of consciousness, with a Glasgow Coma Scale (GCS) of 8. The rest of his examination revealed a left gaze deviation, and he had increased tone globally. His left patellar tendon reflex was absent and he had bilateral up-going plantar reflexes. He was hypertensive, with a systolic blood pressure of 200mmHg. His initial blood investigations revealed a raised urea level, but the rest of his investigations were unremarkable (Table 1). A head computed tomography (CT) with angiography revealed marked posterior fossa oedema resulting in tonsillar herniation and mild hydrocephalus, but no vascular abnormalities. 

**Table 1:** Initial blood results on admission

Test	Result	Unit	Reference Range
Urea	10.7	mmol/L	2.5-7.8
Creatinine	80	mmol/L	64-104
Sodium	144	mmol/L	133-146
Potassium	4.1	mmol/L	3.5-5.3
Adjusted Calcium	2.31	mmol/L	2.2-2.6
Haemoglobin	131	g/L	130-175
White Cell Count	9.0	10^9^/L	3.6-11.0

**Figure 1:**
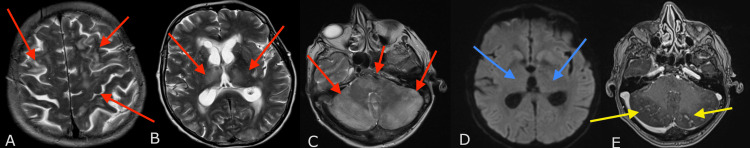
Magnetic resonance imaging of the brain (A)-(C): T2 weighted images. Red arrows show diffuse cortical and subcortical bilateral symmetrical abnormalities in the fronto-parietal and occipital lobes, thalami, brainstem, and cerebellum with mass effect, effaced fourth ventricle and mild hydrocephalus. (D): DWI image: Normal. No restricted diffusion in the thalamus (as shown by blue arrows) or cortical/subcortical abnormalities. (E): linear and punctate enhancement in the cerebellum as shown by the yellow arrows.

The patient was admitted to the intensive care unit for close observation and airway support. Magnetic resonance imaging (MRI) study revealed widespread symmetric T2/FLAIR changes in the thalami, brainstem, and cerebellum, as well as bilateral cortical/subcortical frontal and occipital lobes. The affected regions demonstrated florid punctate enhancement but without restricted diffusion. Multiple posterior fossa-predominant microhemorrhages were also present. There was a significant mass effect with effaced fourth ventricle, tonsillar herniation, and mild supratentorial hydrocephalus (Figure 1).

In the intensive care unit, he was intubated and ventilated. He was started on ceftriaxone, amoxicillin, aciclovir, and high-dose methylprednisolone. Neuroprotective measures were undertaken and mannitol was initiated to manage raised intracranial pressure. He was then transferred to a tertiary center for a lumbar puncture, as this carried a risk of further tonsillar herniation due to elevated intracranial pressure (ICP), and consideration of further neurosurgical intervention if needed. The patient's electroencephalogram was normal. CSF analysis showed elevated protein levels, but glucose and white cell counts were within normal limits (Table 2). The vasculitic screening, which includes Antinuclear antibody (ANA), Rheumatoid Factor, Complement C3, Complement C4, Cryoglobulins, Immunoglobulins (IgG,IgA, and IgM) and Anti-Neutrophil Cytoplasmic Antibody (ANCA), and autoimmune screen for Aquaporin-4 Antibodies, Anti-NMDA receptor Antibody, Anti-GABA B Receptor Antibody, Anti-AMPA Receptor Antibody, Anti CASPR2 Antibodies, Myelin Associated Glycoprotein Antibody, Anti-GD-1 Antibody, Anti-GD-2 Antibody, Myelin Oligodendrocyte Glycoprotein Antibody, and Anti-GQ 1b Antibody were all negative. 

**Table 2:** CSF Analysis Results

Test	Result	Reference Range
CSF White Blood Cells	<1x10^6^/L-Predominantly lymphocytes	0-5x10^6^/L- Predominantly Lymphocytes
CSF Protein	1.77 g/L	0.15-0.45 g/L
CSF glucose	4.0 mmol/L	2.8-4.2 mmol/L

His paraneoplastic autoimmune panel (Anti-Hu, Anti-Yo, Anti-Ri, MA2, and CRMP5 Antibodies) was negative. A CT of the thorax, abdomen, and pelvis did not reveal any underlying malignancy. The initial viral PCRs for herpes simplex virus, varicella-zoster virus, and cytomegalovirus were negative. The main differentials at that time included acute disseminated encephalomyelitis (ADEM) and viral rhomboencephalitis. However, his extended infectious disease screening (mumps, parechovirus, enterovirus, hepatitis A-E viruses, leptospirosis, *Coxiella burnetii*, leptospirosis, rickettsia, Lyme disease, human immunodeficiency virus, syphilis, malaria, dengue, Japanese encephalitis virus, Zika virus, and flavivirus) was negative.

**Figure 2:**
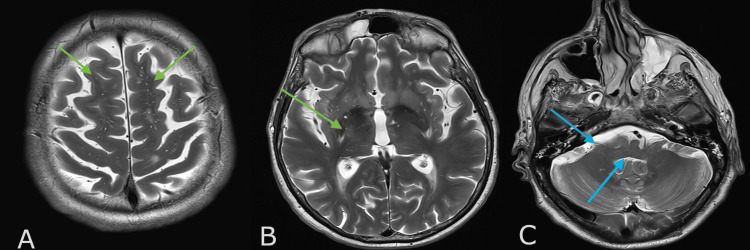
Magnetic resonance imaging of the brain following stabilisation of blood pressure (A), (B), and (C): T2 Weighted images showing near complete resolution of the cortical/subcortical signal in the cerebral hemisphere (green arrows in Figure A), thalami (green arrows in Figure B), brainstem, and cerebellum. In comparison to the previous images, CSF spaces and cisterns were normal with no mass effect (blue arrows in Figure C).

As his extensive infectious screen and autoimmune panel came back negative, our focus shifted to his persistently high blood pressure. Amlodipine, Doxazocin, and Labetalol were added to lower his blood pressure to a systolic of <140mmHg. After four days of intensive care unit stay, blood pressure control was achieved and the patient improved clinically. A repeat MRI showed marked improvement of the supratentorial and infratentorial T2/FLAIR changes and post-contrast enhancement with near complete resolution of the mass effect (Figure 2). 

## Discussion

Our case depicts the unusual presentation of a patient with a history of collapse with a decreased level of consciousness and atypical neuroradiological features. As mentioned in our introduction, PRES usually presents with symptoms such as headaches, visual disturbances, seizures, and altered mental status. Collapse or sudden loss of consciousness are rarer manifestations of this condition [1-2]. This presentation could be explained by the imaging findings of brainstem involvement and posterior fossa oedema leading to tonsillar herniation, which further highlights the atypical nature of our patient's presentation.

The key MRI feature of PRES includes vasogenic oedema, which presents as hyperintense areas on T2 and FLAIR sequences. These lesions are typically symmetrical, bilateral, and characteristically involve the parietal and occipital lobes [5-6]. Other atypical regions involving the brainstem and basal ganglia have also been described in the literature. However, these are rare and account for 10-20% of all cases [6-7]. In making the diagnosis of PRES, other neurological pathologies also present with widespread T2 abnormalities, such as an ischaemic event, encephalitis, demyelinating conditions, vasculitis, and autoimmune disorders, should be excluded [4,6,7].

Diffusion-weighted Imaging (DWI) helps to differentiate vasogenic oedema from cytotoxic oedema, as the latter displays restricted diffusion on DWI [2]. In our case, the lack of restricted diffusion helped to rule out an ischaemic event as the cause of widespread T2 abnormalities. Given the patient's travel history, MRI findings, and the negative autoimmune panel and paraneoplastic screen, the early differentials primarily included ADEM and viral rhomboencephalitis. ADEM, even though rare in adults, most commonly presents with several asymmetric white matter lesions, which are hyperintense on T2 and FLAIR sequences. These lesions can affect both white and grey matter, including the thalamus and basal ganglia [8]. MRI features of viral encephalitis, such as Japanese encephalitis, have an anatomical predisposition for the thalamus and basal ganglia, while cerebral white matter and brainstem involvement are uncommon [9]. 

The atypical clinical presentation, coupled with the patient’s travel history, made the diagnosis of PRES challenging. In addition to that, hypertension was undervalued and not considered during the initial diagnosis. Multiple agents were needed to regulate our patient's persistently elevated blood pressure. As soon as the blood pressure was stabilised, the general condition of our patient improved and a diagnosis of PRES was made. 

One of the differentials for PRES includes reversible cerebral vasoconstriction syndrome (RCVS). RCVS tends to present with thunderclap headaches, focal neurological deficits, and seizures. The clinical presentation of this condition can overlap with PRES. However, the hallmark of RCVS includes cerebral vasoconstriction, which can be assessed by cerebral angiography or magnetic resonance angiography. In our case, the normal cerebral angiography and typical MRI findings of vasogenic oedema helped to rule out RCVS in favour of PRES [4,10]. 

With PRES, acute severe hypertension is the most common triggering factor, with peak systolic blood pressures of 170 mmHg to 190 mmHg being classically described in the literature [5]. Sharma et al. reported a similar case of a 40-year-old gentleman who presented with abnormal eye movements and confusion. He had MRI findings suggestive of PRES and his systolic blood pressure was 234 mmHg on admission. He was started on anti-hypertensive therapy and hemodialysis sessions, which lowered his blood pressure to 160/110 mmHg and led to a complete reversal of his symptoms [11]. 

The mainstay of the management of PRES includes an early diagnosis and control of risk factors such as hypertension [12]. As of now, there are no specific regimens for the acute management of hypertension in PRES [5]. However, blood pressure should be lowered gradually as an aggressive reduction of blood pressure can lead to hypo-perfusion and increase the risk of cerebral ischaemia. An initial blood pressure reduction of 20-25% in the first few hours is recommended and the target mean arterial pressure for this condition should be between 105-125 mmHg [3]. If an early diagnosis is made, most patients can recover fully within two weeks, which highlights the need for a prompt diagnosis [13]. 

## Conclusions

Clinicians should be aware of the atypical presentations of PRES, such as thalami, brainstem, and cerebellar involvement with posterior fossa oedema, to accurately diagnose PRES. We also want to highlight to the neurology community that when faced with complex symptoms, common conditions need to be excluded first, even if confounding factors are present in the patient history. In our case, travel history was a significant confounding factor, and this, coupled with the atypical MRI features, made the diagnosis of PRES challenging. Accelerated hypertension, in the context of clinico-radiological neurological findings without systemic involvement, should raise the suspicion of PRES.
